# Cucurbitaceae genome evolution, gene function, and molecular breeding

**DOI:** 10.1093/hr/uhab057

**Published:** 2022-01-19

**Authors:** Lili Ma, Qing Wang, Yanyan Zheng, Jing Guo, Shuzhi Yuan, Anzhen Fu, Chunmei Bai, Xiaoyan Zhao, Shufang Zheng, Changlong Wen, Shaogui Guo, Lipu Gao, Donald Grierson, Jinhua Zuo, Yong Xu

**Affiliations:** 1 Key Laboratory of Vegetable Postharvest Processing, Ministry of Agriculture, Beijing Key Laboratory of Fruits and Vegetable Storage and Processing, Key Laboratory of Biology and Genetic Improvement of Horticultural Crops (North China) of Ministry of Agriculture, Key Laboratory of Urban Agriculture (North) of Ministry of Agriculture, Beijing Vegetable Research Center, Institute of Agro-Products Processing and Food Nutrition, Beijing Academy of Agriculture and Forestry Sciences, Beijing 100097, China; 2 Department of Food Biotechnology, College of Food Science and Nutritional Engineering, China Agricultural University, Beijing 100083, China; 3 Ministry of Education Key Laboratory of Biodiversity Sciences and Ecological Engineering and State Key Laboratory of Genetic Engineering, Institute of Biodiversity Sciences and Institute of Plant Biology, School of Life Sciences, Fudan University, 2005 Songhu Road, Shanghai 200438, China; 4 School of Biosciences, University of Nottingham, Sutton Bonington Campus, Loughborough, Leicestershire, LE12 5RD, United Kingdom

## Abstract

Cucurbitaceae is one of the most genetically diverse plant families in the world. Many of them are important vegetables or medicinal plants and are widely distributed worldwide. The rapid development of sequencing technologies and bioinformatic algorithms has enabled the generation of genome sequences of numerous important Cucurbitaceae species. This has greatly facilitated research on gene identification, genome evolution, genetic variation, and molecular breeding of cucurbit crops. So far, genome sequences of 18 different cucurbit species belonging to tribes Benincaseae, Cucurbiteae, Sicyoeae, Momordiceae, and Siraitieae have been deciphered. This review summarizes the genome sequence information, evolutionary relationships, and functional genes associated with important agronomic traits (e.g. fruit quality). The progress of molecular breeding in cucurbit crops and prospects for future applications of Cucurbitaceae genome information are also discussed.

## Introduction

Cucurbitaceae is the second largest fruit and vegetable family and its members are among the most important edible plants in the world, next only to Solanaceae [1, 2]. The family contains ~115 genera and 960 species, which are mostly herbaceous annual vines or perennial lianas, often with tendrils [3]. They can be monoecious or dioecious (occasionally hermaphrodite) and are mainly distributed in tropical and subtropical zones, rarely in temperate zones [3]. A characteristic feature of the Cucurbitaceae is the existence of bicollateral vascular bundles where the phloem is present on both the outer and the inner side of the xylem [4]. Cucurbits frequently contain cucurbitacin, which is the main substance causing the bitter taste [5]. The family Cucurbitaceae contains a variety of vegetables or fruit crops, which are of great significance to the global or local economy. The vegetables include cucumber (*Cucumis sativus*), zucchini (*Cucurbita pepo*), pumpkin (*Cucurbita maxima*, *Cucurbita moschata*, and *Cucurbita argyrosperma*), wax gourd (*Benincasa hispida*), bottle gourd (*Lagenaria siceraria*), bitter gourd (*Momordica charantia*), ridge gourd (*Luffa acutangula*), sponge gourd (*Luffa cylindrica*), chayote (*Sechium edule*), and snake gourd (*Trichosanthes anguina*), and the fruits include melon (*Cucumis melo*), horned cucumber (*Cucumis metuliferus*), watermelon (*Citrullus lanatus*), and luo-han-guo (*Siraitia grosvenorii*) [2, 3]. Among them, bitter gourd and luo-han-guo both have rich edible and medicinal value [6] and snake gourd and bottle gourd can be used as food and ornaments [7, 8].

Recently, thanks to the rapid advances in sequencing technologies and bioinformatic algorithms, the application of whole-genome sequencing technology in biology has become more and more common [9]. Due to the high cost and low throughput of Sanger sequencing, the initial genome sequencing work was limited to few plant species, mainly model species such as *Arabidopsis thaliana* [10] and *Oryza sativa* [11]. The first Cucurbitaceae crop genome, that of cucumber, was sequenced using Sanger and next-generation Illumina sequencing technologies and released in 2009 [12]. With the emergence of next-generation sequencing, the cost of sequencing was greatly reduced and efficiency was substantially improved, making possible whole-genome sequencing of many commercially important plants in addition to model organisms. Most importantly, third-generation sequencing technologies (e.g. Oxford Nanopore and Pacific Biosciences) that produce longer reads instead of short reads, chromosome conformation capture techniques, and novel computational methods have together improved the completeness and contiguity of genome assemblies [13, 14]. To date, a number of Cucurbitaceae genomes have been assembled, including *Cucumis sativus* [12, 15–19], *Cucumis melo* [20–25], *Cucumis hystrix* [26], *Cucumis × hystrix* [27], *Cucumis metuliferus* [28], *Cucurbita pepo* [29], *Luffa siceraria* [30], *C.itrullus lanatus* [31–33], *Cucurbita moschata*, *Cucurbita maxima* [34], *Cucurbita argyrosperma* [35, 36], *B. hispida* [37], *Luffa cylindrica* [38–40], *Luffa acutangula* [40], *M. charantia* [6, 41, 42], *Siraitia grosvenorii* [43, 44], *T. anguina* [45], and *Sechium edule* [46].

The completion of genome sequencing of several Cucurbitaceae crops has injected new impetus into the study of genome structure and functional evolution of Cucurbitaceae. This is of great practical significance for further study of the Cucurbitaceae at the genomic level, understanding biological mechanisms, and improving the quality of Cucurbitaceae crops at the molecular level. This review summarizes the findings of whole-genome sequencing and resequencing of Cucurbitaceae plants, which have provided basic data for genome-wide studies of important Cucurbitaceae plants, and discusses the molecular regulation of important traits and prospects for their application to improving fruit quality and promoting plant breeding.

## Whole-genome sequencing of cucurbit crops

As sequencing technologies have developed rapidly, the experimental data and genome sequences of some species have been reinterpreted or revised and improved using new technologies, and this has enabled more complete genome assemblies to be constructed [16, 24]. The cucumber genome sequence [12] was quickly followed by melon [23] and watermelon [31] sequences. Many improved or new genome assemblies of Cucurbitaceae species have been produced during the past 5 years (Table 1). The assembled genome sizes of Cucurbitaceae crops range from 204.8 to 919.76 Mb with a scaffold N50 ranging from 620.88 kb to 82.12 Mb.

**Table 1:** Overview of genome sequences of Cucurbitaceae plant s.

**Date**	**Sequencing technologies**	**Cucurbitaceae species**	**Accession name**	**Chromosome number (*n*)**	**Genome size (Mb)**	**Contig N50 (kb)**	**Scaffold N50 (Mb)**	**Anchored (Mb)**	**Oriented (Mb)**	**Complete BUSCOs (%)**	**Repetitive sequences (%)**	**Protein-coding genes**
2009	Sanger and Illumina	Cucumber [12] (*Cucumis sativus* var. *sativus*)	‘Chinese long’ inbred line 9930	7	243.50	19.80	1.14	177.30 (72.80%)			24.00	26 682
2011	454, Sanger-Celera/Arachne	Cucumber [17] (*Cucumis sativus* var. *sativus*)	B10	7	323.00	23.28	0.32				16.09	26 587
2012	454 pyrosequencing and Sanger	Melon [23] (*Cucumis melo*)	Double-haploid line DHL92	12	375.00	18.20	4.68	316.30 (87.52%)	291.90 (80.77%)		19.70	27 427
2012		Cucumber [18] (*Cucumis sativus* var. *sativus*)	Gy14	7	193.00			173.10 (89.90%)				
2013	Illumina	Watermelon ^31^ (*Citrullus lanatus* ssp. *vulgaris*)	Inbred line 97 103	11	353.50	26.38	2.38	330.00 (93.48%)			45.20	23 440
2013	Illumina	Wild cucumber [15] (*Cucumis sativus* var. *hardwickii*)	PI183967 (CG0002)	7	204.80	119.00	4.20				31.10	23 836
2016	Illumina	Luo-han-guo [43] (*Siraitia grosvenorii*)		14	420.15	34.15	0.10					
2017	Illumina	Bottle gourd [30] (*Lagenaria siceraria*)	Inbred line USVL1VR-Ls	11	313.40	28.30	8.70	308.00 (98.30%)	284.00 (90.60%)	95.40	46.90	22 472
2017	Illumina	Bitter gourd [6] (*Momordica charantia*)	Inbred line OHB3-1	11	285.50	21.90	1.10	172.00 (60.20%)		95.80	15.30	45 859
2017	Illumina	Squash [34] (*Cucurbita maxima*)	Cultivar ‘Rimu’	20	271.40	40.70	3.70	211.40 (77.90%)	201.20 (74.14%)	95.50	40.30	32 076
2017	Illumina	Pumpkin [34] (*Cucurbita moschata*)	Cultivar ‘Rifu’	20	269.90	40.50	4.00	238.50 (88.40%)	221.30 (82.00%)	95.90	40.60	32 205
2018	Illumina HiSeq 2000	Zucchini [29] (*Cucurbita pepo* ssp. *pepo*)	MU-CU-16	20	263.00	110.00	1.80	214.10 (81.40%)		92.10	37.80	27 870
2018	Illumina, SMRT	Luo-han-guo [44] (*Siraitia grosvenorii*)	Variety ‘Qingpiguo’	14	469.50	432.38				89.20	51.14	30 565
2019	Illumina	Melon [22] (*Cucumis melo* var. *makuwa*)	‘Chang Bougi’	12	344.00	15.00	1.00				52.00	36 235
2019	Illumina	Melon [22] (*Cucumis melo* var. *Makuwa*)	SW3	12	354.00	25.00	1.60				54.00	38 173
2019	PacBio, BioNano, Hi-C and genetic maps	Watermelon [32] (*Citrullus lanatus*)	Inbred line 97 103	11	365.10	2300.00	21.90	362.70 (99.30%)		96.80	55.55	22 596
2019	Illumina	Watermelon [33] (*Citrullus lanatus* ssp. *vulgaris*)	‘Charleston Gray’	11	396.40	36.70	7.47	382.50 (96.50%)	379.20 (95.66%)	91.80	49.17	22 546
2019	Illumina, SMRT sequencing and Hi-C	Melon [24] (*Cucumis melo* var. *inodorus*)	‘Payzawat’	12	386.00	2860.00		380.79 (98.53%)	363.76 (95.53%)	92.78	49.80	22 924
2019	Illumina, Illumina MiSeq, and PacBio RS II	Silver-seed gourd [35] (*Cucurbita argyrosperma* ssp. *argyrosperma*)	SMH-JMG-627	20	228.80	463.40	0.62			93.20	34.10	28 298
2019	PacBio, 10X Genomics, and Hi-C technologies	Cucumber [16] (*Cucumis sativus* var. *sativus*)	‘Chinese long’ inbred line 9930	7	226.20	8900.00	11.50	211.00 (93.30%)			36.43	24 317
2019	Illumina and PacBio	Wax gourd [37] (*Benincasa hispida*)	Inbred line B227	12	913.00	68.50	3.40	859.00 (94.10%)		91.00	75.50	27 467
2020	Illumina and PacBio	Cucumber [19] (*Cucumis sativus* var. *sativus*)	B10	7	342.29	858.00				91.30		27 271
2020	PacBio	Melon [20] (*Cucumis melo*)	DHL92	12	357.64	714.00		343.00 (96.00%)		94.80		29 980
2020	ONT, Bionano optical map, Illumina HiSeq, mate pair, and linkage map information	Melon [21] (*Cucumis melo* var. *reticulatus*)	‘Harukei-3’	12	378.00	8600.00	17.50			95.30	55.82	33 829
2020	Nanopore and Hi-C	Snake gourd [45] (*Trichosanthes anguina*)		11	919.76	20110.00	82.12	918.80 (99.89%)		95.38	80.03	22 874
2020	PacBio and Hi-C	Sponge gourd [38] (*Luffa cylindrica*)		13	669.00	5000.00	53.00			91.60	62.18	31 661
2020	Illumina, PacBio, 10× Genomics and Hi-C	Sponge gourd [39] (*L. cylindrica*)	Inbred line P93075	13	656.19	8800.00	48.76				63.81	25 508
2020	SMRT and Chicago/Hi-C	Ridge gourd [39] (*Luffa acutangula*)	Inbred line AG-4	13	734.60		0.79			92.70	62.17	32 233
2020	SMRT and Chicago/Hi-C	Sponge gourd [39] (*Luffa cylindrica*)	Inbred line SO-3	13	689.80		0.58			93.00	56.78	43 828
2020	PacBio and Hi-C	Bitter gourd [41] (*M. charantia*)	OHB3-1	11	302.99	9898.00	25.37	291.70 (96.27%)		96.40	52.52	
2020	Illumina	Bitter gourd [42] (*M. charantia*)	‘Dali-11’	11	293.60	62.60	3.30	251.30 (85.50%)		96.70	41.50	26 427
2020	Illumina	Bitter gourd [42] (*M. charantia*)	TR	11	296.30	16.10	0.60			97.10	39.90	28 827
2021	Illumina and PacBio	Silver-seed gourd [36] (*Cucurbita argyrosperma* ssp. *sororia*)		20	255.20	1205.50	12.10	98.80		92.80		30 592
2021	Illumina and PacBio	Silver-seed gourd [36] (*C. argyrosperma* ssp. *argyrosperma*)	SMH-JMG-627	20	231.60	447.00	11.70	99.97		93.20		27 998
2021	Illumina	*Cucumis hystrix* [26]		12	297.50	220.95	14.06	268.90 (90.4%)	262.40 (88.2%)	93.50		23 864
2021	Illumina, SMRT, Hi-C, and BioNano optical mapping	*Cucumis × hytivus* [27]		19	540.75	6596.00	27.20	525.78 (97.23%)	490.71 (93.33%)		50.98	45 687
2021	SMRT and Hi-C	*Cucumis metuliferus* [28]	CM27 (PI 482460)	12	329.00	2900.00		316.82 (97.99%)	316.82 (97.99%)	93.54	42.63	29 214
2021	SMRT and Hi-C	Melon [28] (*Cucumis melo* ssp. *agrestis*)	IVF77	12	364.00	490.00		339.72 (96.31%)	339.72 (96.31%)	93.26	51.11	27 073
2021	Illumina, SMRT	Bottle gourd [8] (*Lagenaria siceraria*)	‘Hangzhou Gourd’	11	297.00	11200.00	28.40			95.50	44.99	23 541
2021	Nanopore and Hi-C	Chayote [46] (*Sechium edule*)		14	608.17	8400.00	46.56	606.42 (99.71%)	598.48 (98.41%)	95.62	65.94	28 237

BUSCOs, Benchmarking Universal Single-Copy Orthologs; SMRT, single-molecule real-time; Hi-C, high-throughput chromosome conformation capture.

According to the reported syntenic relationships among genomes of cucurbits, including melon (*n* = 12), cucumber (*n* = 7), wax gourd (*n* = 12), bottle gourd (*n* = 11), watermelon (*n* = 11), and pumpkin (*n* = 20), it is inferred that the ancestral cucurbit protochromosome number was 15 and the most ancestral state is preserved in the wax gourd genomes among these species [37]. Collinearity analysis showed that the seven chromosomes of melon are derived directly from the ancestral ones, which is the most preserved cucurbit ancestral karyotype after that of the wax gourd, and the bottle gourd genome is the third best preserved ancestral cucurbit karyotype after those of the wax gourd and melon [30, 37]. Researchers have suggested that the bottle gourd chromosomes derived from the ancestral Cucurbitaceae karyotypes through 19 chromosomal fissions and 20 fusions [30]. The modern 11-chromosome structure of watermelon evolved from the ancestral Cucurbitaceae karyotype (12 chromosomes) through 27 fissions and 28 fusions, which indicates that the watermelon genome has undergone more rearrangements than that of the bottle gourd [30]. An extensive chromosomal rearrangement has also occurred in the zucchini genome [29], and the complicated syntenic patterns have unveiled the great complexity of chromosomal evolution and rearrangements in important Cucurbitaceae crops. In pumpkin, six chromosomes remained in the ancestral karyotype state, whereas all chromosomes of cucumber and watermelon were formed through many fusions and fissions [37]. The collinearity analysis showed that, of the five chromosomes in cucumber, each had a one-to-two syntenic relationship with 10 of the melon chromosomes [12]. Four chromosomes in snake gourd each virtually had a one-to-one syntenic relationship with sponge gourd chromosomes [45], which indicates that they are closely related to each other. Overall, this information is of fundamental importance for comparative genomics in cucurbits.

## Genome evolution

Besides the whole-genome triplication event (gamma) shared by all eudicots, it seems that at least four additional whole-genome duplication (WGD) events occurred during the evolution of Cucurbitaceae plants [2]. An early large-scale duplication event (CucWGD1), a cucurbit-common tetraploidization at the origin of the Cucurbitaceae family, has been identified, which occurred shortly after the gamma event (115–130 Mya) [2, 46, 47]. Moreover, three relatively recent WGDs have been identified within three tribes [2]. The tribe Cucurbiteae probably experienced one WGD at its origin (CucWGD2) [34]. Several studies have shown that zucchini (*Cucurbita pepo*), pumpkin (*Cucurbita moschata* and *Cucurbita maxima*), and silver-seed gourd (*Cucurbita argyrosperma*) from the tribe Cucurbiteae underwent WGD events [29, 34, 35]. In addition, several members of the tribe Sicyoeae also exhibit evidence for one WGD event (CucWGD3) [2]. One recent WGD event occurred in chayote (*Sechium edule*) of the tribe Sicyoeae at about 25 ± 4 Mya [46]. CucWGD4 is likely shared by the members of *Hemsleya* in the Gomphogyneae tribe [2].

**Figure 1:**
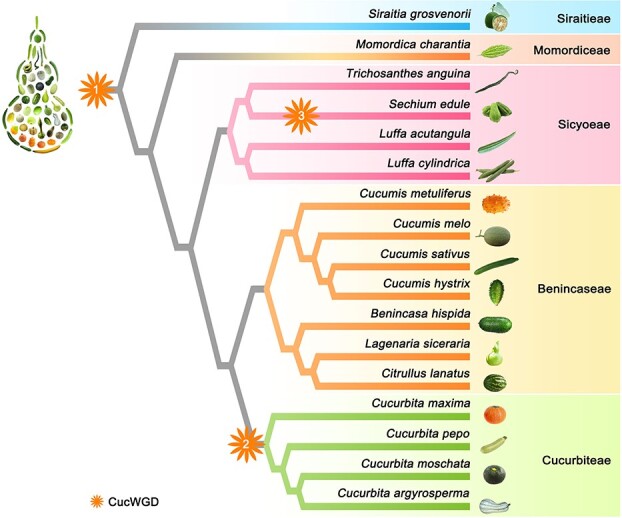
Phylogenetic tree based on the reported phylogenetic relationships of 17 Cucurbitaceae species. Orange stars indicate cucurbit-specific WGD (CucWGD) events. Tribes are listed to the right of the tree.

According to the evolutionary relationship among Cucurbitaceae [26, 28, 30, 34, 37, 39, 42, 44–46], we summarized the phylogeny of 17 sequenced Cucurbitaceae species (*Cucumis* × *hytivus* is not included) by integrating relevant information (Fig. 1). Phylogenetic analysis indicates that a variety of fruit and vegetable Cucurbitaceae crops emerged with different shapes due to species differentiation after the first shared WGD event. Among these 17 species, the first divergent species appears to be luo-han-guo followed by bitter gourd [2, 34–37]. The Sicyoeae branch containing sponge gourd, ridge gourd, snake gourd, and chayote diverged sequentially [45, 46]. Species belonging to Benincaseae and Cucurbiteae form the sister clades. The Benincaseae tribe is represented by the four successively divergent genera of *Cucumis*, *Benincasa*, *Lagenaria*, and *Citrullus* [34, 37], and the Cucurbiteae tribe is represented by four *Cucurbita* species with the sister pairs of *Cucurbita moschata* and *Cucurbita argyrosperma* grouped with *Cucurbita pepo* and *Cucurbita maxima* in succession [34, 35] (Fig. 1). Although the divergence times among these cucurbit crops have been estimated using a Bayesian method [2], the exact divergence time of each Cucurbitaceae species remains unclear.

The history of the speciation events has been reported in several studies on genomic research and sometimes there is a conflict of the estimated species divergence time [30, 34–37, 45, 46], which may be affected by the species representativeness, method, fossils, and confidence interval used in estimating the time. For example, the divergence between cucumber and melon has been variously estimated at 8.4–11.8 (the median value is 10.1) Mya [23, 30, 37, 48]. However, according to the research of Ma *et al*. [45], Fu *et al*. [46], and Sun *et al*. [34], the two species (cucumber and melon) diverged ~5–12, 4–14, and 6.06–6.94 Mya, respectively. Therefore, estimates of the divergence between cucumber and melon range from about 4 to 14 Mya. This information is summarized in Table 2.

**Table 2:** Reported estimates of divergence and evolution of members of the Cucurbitaceae.

**Cucurbitaceae species**	**Divergence time (Mya)**	**Reference**	**Comprehensive viewpoint (Mya)**
*Cucumis sativus* and *Cucumis melo*	5–12	45	4–14
	8.4–11.8	30	
	4–14	46	
	10.1	37	
	6.06–6.94	34	
	12.59	28	
	9	44	
	8.2–10.0	42	
	9.63	40	
	6.8	39	
	9.6	26	
	10	30	
*Cucumis hystrix* and *Cucumis sativus*	4.5	26	4.5
*Cucumis metuliferus* and *Cucumis melo*	17.85	28	17.85
*Citrullus lanatus* and *Lagenaria siceraria*	14–27	45	10–30
	10.4–14.6	30	
	12–30	46	
	13.8	37	
	18.34–19.75	34	
	16	28	
	13.0–15.7	42	
	15.11	40	
	14.9	39	
	21.4	26	
	10–14	30	
*B. hispida* and *Citrullus lanatus*/*Lagenaria siceraria*	16.3	37	16.3–18.1
	18.1	40	
	16.8	39	
*Siraitia grosvenorii* and *Citrullus lanatus*	40.9	44	40.9
*Momordica charantia* and other	48–77	45	29.2–96
	29.2–41	30	
	39–96	46	
	36.1	37	
	44.1 ± 14	35	
	34.94–37.24	34	
	33.3–40.4	42	
	44.06	40	
	41.6	39	
	49.8	26	
*Cucurbita maxima* and *Cucurbita moschata*	3.04–3.84	34	3.04–7.3
	5.3–7.3	42	
	4.81	40	
*Cucurbita maxima* and *Cucurbita pepo*	13.9	28	13.9
*Cucurbita moschata* and *Cucurbita pepo*	3.9–5.4	42	3–16
	3–16	46	
	3–13	45	
	6.3	39	
*Cucurbita moschata* and *Cucurbita argyrosperma*	3.98 ± 1.7	35	3.98 ± 1.7
*T. anguina* and *Sechium edule*	27–45	46	27–45
*Luffa acutangula* and *Luffa cylindrica*	7.97	40	7.97
*Luffa cylindrica* and *T. anguina*	33–47	45	29–55
	29–55	46	

## Genes associated with important agronomic traits

With the development of the whole-genome sequences of Cucurbitaceae, a large number of coding genes have been annotated and genes related to fruit and vegetable quality traits have begun to be identified. A wide range of important phenotypic and agronomic traits of Cucurbitaceae plants include pathogen resistance, fruit size, mass, color, texture, length, shape, rind form, ripening behavior, sugar content, bitterness, flavor and aroma, sex determination, and tendrils [12, 24, 32]. Population analysis and genome-wide association studies (GWAS) on diverse species accessions has contributed to the identification of a number of candidate genes controlling desirable fruit and vegetable traits [24]. This provides information for effective breeding strategies and is conducive to the development of high-quality, resilient elite cultivars of Cucurbitaceae species [23, 25].

### Resistance genes

Plant resistance (R) genes are among the most important targets for plant breeding programs and have been the object of intense research. R genes can activate plant defense systems to restrict pathogen invasion and improve plant resistance against major diseases [49]. The major resistance genes have been identified in various Cucurbitaceae species. Among these genes, those encoding the nucleotide-binding site leucine-rich repeat (NBS-LRR) proteins are related to effector-triggered immunity, which is a significant component of plant disease resistance [50]. A total of 44 *NBS-LRR* (*NLR*) genes, consisting of 26 *coiled-coil* (*CC*)-*NBS-LRR* (*CNL*) and 18 Toll interleukin receptor (*TIR*)-*NBS-LRR* (*TNL*) genes, have been identified in watermelon [31]. The number of *NLR* genes identified in cucumber, melon, wax gourd, *Cucurbita maxima, Cucurbita moschata*, and bitter gourd are 74, 84, 82, 30, 57, and 78, respectively [34, 37].

Research on the aphid resistance of cucumber cultivar ‘EP6392’ showed that 8 of the 49 DEGs may be relevant to aphid resistance [51]. The volatile (*E*,*Z*)-2,6-nonadienal (NDE) is involved in resistance to a number of bacteria and fungi in cucumber [52]; several *EIF4E* and *EIF4G* genes were found to be resistant to plant RNA virus infections, and two *At* (glyoxylate aminotransferase) gene homologs conferring potential resistance to downy mildew have also been identified [12]. Interestingly, an *EIF4E* gene found in melon mediates recessive resistance against melon necrotic spot virus [53–55], and the increased expression of two glyoxylate aminotransferase (*At1* and *At2*) genes was found in wild melon genotypes, which may contribute to their resistance to downy mildew [56].

The most prevalent viruses that have a significant impact on the production of cucurbit crops are aphid-transmitted viruses in the Potyviridae family, including papaya ring-spot virus watermelon strain (PRSV-W), zucchini yellow mosaic virus (ZYMV), and watermelon mosaic virus (WMV) [57–64]. Of these, PRSV-W is one of the most destructive viruses that infect cucurbits worldwide [65–67]. The bottle gourd USVL5VR-Ls line is resistant to PRSV-W [68], and resistance is determined by *Prs*, an unidentified dominant monogenic locus [30]. An *NBS-LRR* gene (*RGH10*) was shown to confer PRSV resistance in melon [69]. Research has showed that ethylene signaling may participate in the PRSV resistance mechanism in cucurbits. AP2/ERF transcription factors (TFs) have been reported as the basis of plant defense mechanisms against a wide range of pathogens, including viruses, which makes the *AP2/ERF* gene family a feasible source of candidate genes for *Prs* [70]. In the snake gourd genome, five R genes potentially involved in the plant–pathogen interaction pathway have been identified [45]. Changes in their expression are associated with the changes in resistance during fruit ripening, which may possibly be related to the resistance of snake gourd to pathogens and insects [45].

### Sex determination

In Cucurbitaceae, sex determination is closely related to fruit earliness, yield, and quality [71]. Ethylene stimulates femaleness and is regarded as the main regulatory factor of sex determination [72–74]. Naturally occurring mutations in the genes encoding the corresponding enzymes in the ethylene biosynthesis pathway have a notable impact on sex determination in the Cucurbitaceae [75, 76]. For example, a loss-of-function mutation in a *1-aminocyclopropane-1-carboxylic acid synthase* (*ACS*) gene in melon and cucumber leads to the enhancement of ‘maleness’ [75, 77]. There seem to be similar mechanisms at play in *Cucurbita pepo* [78]. In addition, the ACC oxidase gene *CsACO2* is essential in female flower formation in cucumber and mutations in this gene confer androecy [79]. Studies have also shown that ethylene receptors are implicated in the regulation of zucchini sex determination [80, 81]. Cucumber and melon are often used to study sex expression in Cucurbitaceae plants [12, 23, 82]. Three major sex determination genes, *M*, *F*, and *A*, have been established in cucumber, and shown to be members of the *aminocyclopropane-1-carboxylic acid synthase* (*ACS*) gene family (*CsACS1G* for *F*, *CsACS2* for *M*, and *CsACS11* for *A*) [77, 79, 83–86]. Cucumber has a distinctive genetic system for gynoecious sex expression and contains three genes: *CsACS1*, *CsACS1G*, and *CsMYB* [87–90]. Study has revealed that the *CsACS1G* gene is responsible for production and development of female flowers in cucumber gynoecy conferred by the *F* locus [91]. However, this gynoecy expression system appears to be unstable, which may be due to unequal crossing over at the copy number variation (CNV)-based *femaleness* (*F*) locus [87]. The melon sex determination-related gene *Cm-ACS7* and *ACS11*, and cucumber ortholog *Cs-ACS2*, as well as *Cucurbita pepo* ortholog *CpACS27A,* are crucial regulatory enzymes in the ethylene biosynthetic pathway [77, 92–95]. These genes are vital to the suppression of male organs and development of the female flower [77, 92–95]. In addition, the gynoecious locus *CmWIP1* involved in occurrence of gynoecy in melon has also been found to be implicated in sex determination of cucurbits [96–99]. It has two orthologous genes (*CpWIP1A* and *CpWIP1b*) identified in *Cucurbita pepo* [92]. In addition, auxin can regulate sex expression through stimulating ethylene generation [72–74]. Research has suggested that six auxin-related genes and three short-chain reductase or dehydrogenase genes involved in sex determination have higher expression levels in unisexual flowers of cucumber [12]. The identification and functional analysis of these genes have provided valuable information for the study of sex expression in other Cucurbitaceae plants.

### Fruit color

The diverse color of fruit is determined by the concentrations and compositions of various pigments, mainly chlorophylls and carotenoids, as well as flavonoids (especially chalcones and anthocyanins). Melon rinds have a variety of colors, including green, white, orange, yellow, variegated, and striped [100]. It is known that β-carotene accumulation can contribute to the orange color, and the accumulation of lutein and other carotenoids contributes mainly to the yellow color of fruit [101], while the carotenoid content of white-fleshed melon and watermelon can be low or negligible [102, 103]. A yellow flavonoid pigment, naringenin chalcone, was identified as the major pigment in mature rinds of ‘canary yellow’ type melons [100]. Similarly, the main carotenoids that accumulate in yellow-fleshed watermelon and zucchini are lutein and β-carotene [104].

The key genes known to be implicated in the carotenoid metabolic pathway play important roles in regulating carotenoid accumulation, leading to changes in pigmentation [105]. The *CmPPR1* (*EVM0014144*) gene may affect carotenoid accumulation and flesh color in melon [106–108]. The *CmOr* gene controls β-carotene accumulation, resulting in the orange flesh colors in melon fruit [109], while the identified *MELO3C003097* gene may serve as a strong candidate for the Wf locus controlling white and green melon flesh [25]. Moreover, two peel-color-related candidate genes, *MELO3C003375* and *EVM0012228* (*CmKFB*), have been identified [25]; *CmKFB* genes negatively regulated the accumulation of naringenin chalcone determining the yellow color of melon rind [110]. The flesh color of *Cucurbita moschata* and *Cucurbita maxima* usually appears to be yellow and orange, while zucchini is mainly white and pale yellow [111–114]. *β-Carotene hydrolase* (*CHYB*) and *phytoene synthase* (*PSY*) are two main genes affecting the formation of yellow-fleshed fruit of *Cucurbita moschata*, *Cucurbita maxima*, and *Cucurbita pepo* [113–115], while the *carotenoid cleavage dioxygenases 4* (*CCD4*) gene exerts an important function in the regulation of white pulp in *Cucurbita pepo* [112]. Ripe *M. charantia* fruits had higher carotenoid (mainly β-carotene) concentrations [116]. During fruit ripening, increased expression of *phytoene synthase* (*McPSY*) and *phytoene desaturase* (*McPDS*), associated with carotenoid synthesis, was observed, resulting in carotenoid accumulation in the pericarp and a change of peel color from green to orange [116, 117]. A study in *Cucurbita pepo* showed that the up-regulated expression of several structural genes involved in carotenoid metabolic pathways probably leads to the increased carotenoid accumulation in ripe fruit [92]. It is well known in tomato that *PSY1* is a critically important enzyme that is induced during ripening [118, 119]. In ripening fruit of sweet watermelon, the *PSY1* gene may be involved in the transition from pale-colored to red, orange, or yellow flesh through increasing total carotenoid accumulation [32]. Mutation in *LCYB* may lead to increased lycopene content, since artificial selection of the mutation was shown to be responsible for the red flesh color in most sweet watermelon cultivars [32]. Moreover, *ClTST2*, a sugar transporter gene, was credited with facilitating carotenoid accumulation in watermelon fruit flesh [32]. During flesh color formation, the up-regulated expression of gene *ClPHT4;2* was closely related to increased carotenoid contents in watermelon flesh [107, 120]. In chayote fruit, a number of candidate genes regulating pigment accumulation have also been identified, such as *HCAR* (*7-hydroxymethyl chlorophyll a reductase*), regulating chlorophyll content, and *β-carotene hydroxylase 2* (*CHY2*), *CCD1*, *CCD4*, and *ZEP* [46]*.* These genes may be involved in fruit color production [46]. The up-regulated expression of carotenoid accumulation-related genes may contribute to the increase of carotenoid content, making the fruit turn orange-red after ripening in snake gourd fruit [45].

### Fruit size, shape, and texture

There are many factors that affect the formation of fruit shape, and their interaction and coordination eventually lead to differences in fruit shape. Various studies have reported a variety of classical and newly identified key genes related to fruit shape, mainly including SUN, OFP, WOX, YABBY, AP2, and auxin transporters [8, 121–124]. Apart from these well-known genes, sugar signaling and metabolism have been suggested to be related to cell division and growth, which can influence organ shape [125]. Through GWAS analysis, a strong association signal related to fruit shape in watermelon was identified near the *ClFS1* (*Cla97C03G066390*) gene controlling fruit elongation [126]. In addition, other genes or proteins related to fruit shape are also found in different plants, such as the TONNEAU1 recruiting motif protein (TRM5), the *AP2/ERF transcription factor* (*AP2a*) gene in tomato [127, 128], and the *CAD1* gene belonging to the LRR-RLK family in peach [129].

The fruit shapes of Cucurbitaceae plants are diverse, and some genes controlling their shape variation have been identified. Quantitative trait locus (QTL) analysis for cucumber showed that the round fruit shape in WI7239 is controlled by two QTLs, FS2.1 and FS1.2, containing the tomato homologous genes *SlTRM5* (*CsTRM5*) and *SUN* (*CsSUN25–26-27a*), respectively [128, 130]. The deletion of the rst exon of FS1.2 in cucumbers results in the formation of round fruits [130]. In another study, FS5.2 greatly influenced the formation of round fruit in WI7167 cucumber [130]. Watermelon fruits have three major shapes: elongate (OO), oval (Oo), and spherical (oo), controlled by a single, incompletely dominant gene [126]. A candidate gene, *Cla011257*, on chromosome 3 related to watermelon fruit shape (ClFS1) was identified and results suggested that *Cla011257* might control spherical fruit shape and a deletion of 159 bp in *Cla011257* may lead to elongated fruit in watermelon [126]. The wax gourd fruit shapes are mainly long cylindrical, cylindrical, and round [131]. During ovary formation, the expression levels of *Bch02G016830* (designated *BFS*) in round wax gourd fruit are significantly higher than in long cylindrical fruits [131]. Therefore, *BFS* might be a candidate gene for fruit shape in wax gourds [131]. Variations in *BFS* might slow down cell division at the ovary formation stage and may contribute to the regulation of wax gourd fruit size [131]. In *Cucurbita pepo*, a single gene, *Di*, controls the disk fruit shape, which is dominant over spherical or pear-shaped fruit [132]. In *Cucurbita moschata*, the gene *Bn* controls butternut fruit shape and is dominant to *bn* for crookneck fruit shape [133]. In addition, sex expression has pleiotropic effects on cucumber and melon fruit shape [140, 134]. A 14-bp deletion in *CsACS2*, the candidate gene for the monoecious (m) locus in cucumber, resulted in elongated fruit shape in cucumber [95]. The pleiotropic effect of sex expression on fruit shape is also well established in melon [134].

Plant hormones have been showed to contribute to the regulation of fruit size and development [135]. Ethylene participates in many plant development processes and it serves as a triggering signal to initiate climacteric fruit ripening [136, 137]. The *CpACS27A* gene in *Cucurbita pepo* is the homologous gene of *CmACS7* (*MELO3C015444*) in melon, which is involved in ethylene synthesis and sex determination and also influences fruit length [75, 108]. Auxin plays a critical role in cell expansion during fruit development stages [138–140] and the role of the main regulators of auxin—auxin response factors (ARFs)—in cell division and growth have been well established [140, 141]. A total of 56 *ARF* genes were identified in bitter gourd [142], but in other families the number can vary considerably. It has been suggested that auxin-responsive GH3 family genes, auxin-responsive protein (IAA), and SAUR family proteins may be associated with chayote fruit enlargement [46], and the up-regulated expression of auxin-related genes may be involved in snake gourd fruit elongation [45]. SAUR was reported to be implicated in the regulation of plant growth and development through promoting cell expansion [143–145], *Bhi10G001538* and *Bhi10G000196* may be important candidate genes contributing to large fruit during wax gourd domestication [37], and *Bhi10G000196* is orthologous to the tomato gene *SlFIN* (*Solyc11g064850*) responsible for enlarged tomato fruit [146]. In addition, four WUSCHEL TFs have been identified in *Cucurbita pepo* [92], which affect fruit size [147, 148].

During fruit growth, development and ripening, there are many changes to cell wall structure and properties in cell wall biogenesis and modification, cell expansion, unidirectional elongation, and fruit softening [149, 150]. Numerous different types of cell-wall-modifying enzymes have been identified as being involved in the development and ripening processes of many fruits, including the pectin-modifying enzymes [polygalacturonase (PG), pectinesterase (PE), pectate lyase (PL), and β-galactosidase (β-GAL)] and the hemicellulose/cellulose-modifying enzymes [β-1,4-glucanase, xyloglucan transglycosylase/hydrolase (XTH) and expansin (EXP)], which together lead to changes in fruit texture by regulating the structure of cell wall polymers and influence fruit ripening [137, 151]. The increased expression of β-1,4-glucanase or enhanced enzyme activity is usually associated with fruit softening [75]. In addition, six genes (three *pectinesterase* genes, two *gibberellin 20 oxidase 1-B-like* genes, and one *pectate lyase-like* gene) involved in cell wall biosynthesis have been identified that may play important roles in determining epidermis thickness in the melon [24]. Regulation of the expression of many DEGs related to cell wall modification may be associated with fruit texture changes in snake gourd, including *β-galactosidase 10/5-like*, *cellulose synthase-like protein*, *endoglucanase 10/11/17-like*, *expansin-A4/A10-like*, *β-glucosidase 18-like*, and *pectinesterase 53* [45]. Moreover, polygalacturonase, pectinesterase, and cellulose synthase-like protein B4 may affect cell wall properties and fruit texture during chayote development [46]. Expansins are cell wall proteins regulating cell size and fruit growth in plants, and are also highly expressed during fruit development and ripening [152, 153]. Although they have no catalytic activity, the expansins appear to induce loosening of bonds between cellulose and hemicellulose in the cell wall, leading to ‘polymer creep’ within the cell wall during growth, resulting in cell enlargement or shape change [137]. Also, expansins enable cell expansion and fruit softening by triggering the loosening of the cell wall [154]. Expansin-A12 is thought to be implicated in melon fruit size [24], and expansin-like B1, identified in the chayote fruit, may induce plant cell wall extension, with increased transcripts contributing to rapid fruit enlargement [46].

Other genes involved in cell division and cell cycle regulation can also directly influence the growth rate of plant tissues and determine the final size of plant organs [155]. A total of six DEGs related to the regulation of cell division and the cell cycle were identified in bottle gourd [156]. Furthermore, the study of melon showed that *L-ascorbate oxidase* (*AAO*) could play a role in the late stage of fruit development, associated with the change in fruit size [157]. Differential expression of the gene was also found in *Cucurbita pepo* [92], snake gourd [45], and chayote [46]. In *Cucurbita pepo*, up-regulated expression of the *CpOVATE* gene acting as a repressor of growth was observed in the small-fruit ‘Munchkin’, which showed that *OVATE* plays a key role in shorter fruit [158]. Similarly, the *hexokinase* (*CpHXK-1*) and *CpFW2.2* genes were also found to contribute to a reduction in fruit size [158].

### Fruit taste

There are three major components, including acidity, sugar, and volatile flavor compounds, that together contribute to the overall taste of fleshy fruit [159]. The *PH* gene (*CmPH*) identified in melon has an important regulatory effect on fruit acidity [159], and numerous genes involved in the citrate acid cycle that may influence the accumulation of organic acids have also been identified in melon [160]. The *ClBt* gene in watermelon and *CsBt* in cucumber regulate fruit bitterness [5, 32, 161] and volatile *(E,Z)-2,6-nonadienal* (*NDE*) confers on cucumber its ‘fresh green’ flavor [162], while *CmTHAT1* (thiol acyltransferase, EVM0016460) affects fruit flavor [24,108].

Sugar accumulation is the main factor that contributes to the sweet taste, which is particularly important in the fruit ripening process of melon and watermelon. Two candidate genes, *EVM0015625* and *EVM0019658*, have been suggested to be responsible for sugar accumulation in melon and the β-*glucosidase* and α*-l-fucosidase* 2 genes are related to the synthesis and transportation of sugars [24]. In melon fruit, a total of 63 genes may be involved in the sugar metabolism pathway [23], and enzymes considered to be involved in regulating sugar biosynthesis, unloading, transport, and metabolism processes during watermelon flesh development include neutral invertase, α-galactosidase, sucrose phosphate synthase, insoluble acid invertase, soluble acid invertase, UDP-glucose 4-epimerase, and UDP-galactose/glucose pyrophosphorylase [31]. An alkaline α-*galactosidase* gene (*ClAGA2*) was suggested to be related to the accumulation of sugar in watermelon pulp by promoting the metabolism of raffinose into glucose, fructose, and sucrose [32, 163–165]. The roles of vacuolar sugar transporter *ClVST1*, hexose transporter *ClSWEET3*, and tonoplast sugar transporter *ClTST2* in the sugar accumulation of watermelon fruit are well established [165]. *ClVST1* is responsible for glucose and sucrose efflux and unloading in the watermelon fruit [166]. The key transporter protein *ClTST2* contributes to the accumulation of sucrose, fructose, and glucose in the vacuole of watermelon fruit cells [167]. Their expression levels are positively correlated with watermelon fruit sugar content and their overexpression increased fruit sugar accumulation of watermelon flesh [168]. In addition, the overexpression of an ortholog of *ClTST2* (*CmTST2*) in melon fruit could increase sugar content [168]. TF genes putatively implicated in sugar accumulation include a *bZIP* gene, namely *Cla014572*, which functions as a key regulatory factor of sugar accumulation during fruit development [31, 169]. Further work on the identification, differential expression, and functional analysis of these genes will contribute to the understanding of fruit flavor of Cucurbitaceae plants.

The catabolism of several amino acids plays a central role in the production of aroma compounds in melon [170]. Valine, leucine, and isoleucine are implicated in the biosynthesis of branched-chain esters [171], and tyrosine and phenylalanine participate in the biosynthesis of aromatic esters [172]. Ethylene can enhance the levels of these amino acids to promote synthesis of esters, thus affecting melon flavor [170], and ethylene may also enhance aminotransaminase (AT) activity by increasing the expression of *CmBCAT1* and *CmArAT1*, whose gene products convert branched chain amino acids into aroma volatiles through amino acid aminotransferases [172, 173]. The key role of the two genes in the biosynthesis of melon aroma volatiles is well documented [172]. Sulfur-containing aroma volatiles make an important contribution to the distinctive aroma of melon and other fruits [173] and thioether esters greatly promote the fruity aroma of melon fruit [174, 175]. l-Methionine was postulated to be a precursor of aroma volatiles in melon fruit [175]. Two distinct parallel pathways for l-methionine catabolism, a transamination route involving the action of an l-methionine aminotransferase and a γ-lyase route involving the action of an l-methionine-γ-lyase activity encoded by melon gene *CmMGL* is involved in the formation of melon aroma volatiles [173]. In addition, sulfur-containing esters may also be synthesized from cysteine [170].

The cucurbitacins are plant triterpenoids that form the bitter compounds predominant in the Cucurbitaceae family and impart a bitter taste in cucumber, zucchini, melon, pumpkin, and other plant foods [5, 161, 176]. To date, many cucurbitacins, including cucurbitacins A–L, O–T, and several others, have been discovered in plants (https://en.wikipedia.org/wiki/Cucurbitacin). Several studies have shown that they exhibit wide-ranging pharmacological activities, such as cytotoxic, hepatoprotective, purgative, anti-inflammatory, anti-infectious, antidiabetic, antitumour and anticancer effects [177–180]. In addition, cucurbitacin I can suppress cell motility through interfering indirectly with actin dynamics [181]; cucurbitacin B and cucurbitacin I could be beneficial in suppressing adipocyte differentiation and preventing metabolic diseases [182]; and the efficacy of cucurbitacin R and dihydrocucurbitacin B on the immune system has also been recognized [183].

**Figure 2:**
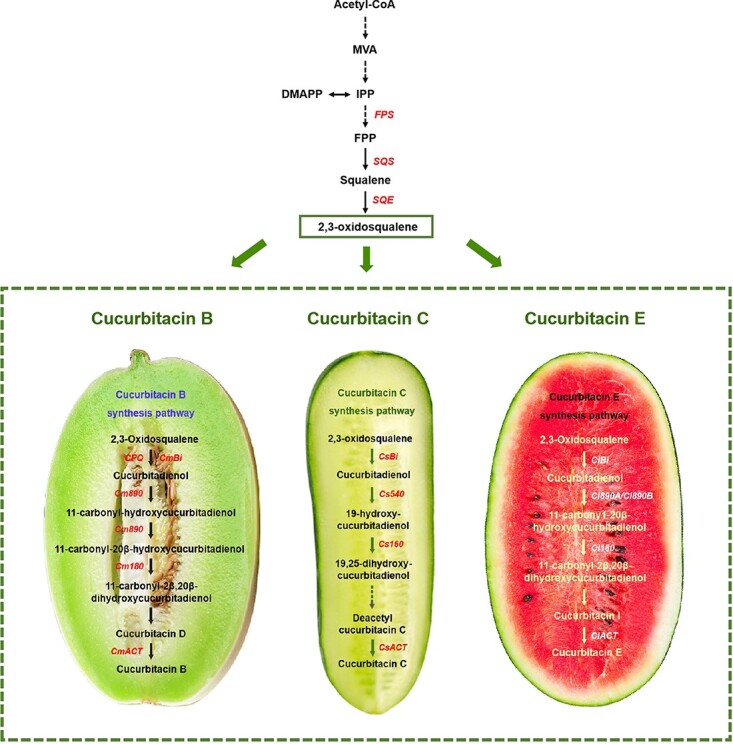
The cucurbitacin biosynthesis pathway in melon, cucumber, and watermelon.

The precursors of cucurbitane triterpenoids are synthesized through the mevalonate pathway [184] and cucurbitadienol is produced by cucurbitadienol synthase, forming the basic skeleton of cucurbitane triterpenoids [185] (Fig. 2). Cucurbitacins C (CuC), B (CuB), and E (CuE) are the main bitter substances isolated from cucumber [5], melon [186], and watermelon [187], respectively. The biosynthesis pathway of CuC has been described by Shang *et al*. [5]; nine CuC biosynthetic enzymes (CsBi, seven CYPs, and CsACT) were identified and four catalytic steps were elucidated. Eight CuB (CmBi, six CYPs, and CmACT) and 10 CuE biosynthetic enzymes (ClBi, 8 CYPs, and ClACT) have also been identified in melon and watermelon, respectively [161]. The cucurbitacin biosynthetic enzymes (Bi, eight CYPs, and ACT) have also been identified in *Luffa acutangula* and *Luffa cylindrica* [39]_._ The biosynthesis pathway of cucurbitane triterpenoid in bitter gourd was reported by Cui *et al*. [42]. The identification of these bitter genes has contributed to understanding the regulatory and biochemical variations of cucurbitacins and provided important information for molecular breeding for taste improvement.

### Transcription factors involved in fruit growth and ripening

Many TF families have important effects on fruit development [188–190]. Myeloblastosis (MYB) proteins are one of the largest TF families in plants and are widely involved in diverse plant-specific processes, such as plant organ development, signal transduction, secondary metabolism, and multiple stress responses [191–194]. In cucumber, two *MYB* genes, *CsMYB6* (*Csa3G824850*) and *CsTRY* (*Csa5G139610*), have been reported to negatively regulate fruit spine or trichome initiation [195]. Other research has shown that the *CsTRY* not only regulates fruit spine or trichome formation, but also plays a negative regulatory role in anthocyanin synthesis [196]. Moreover, *CsMYB60* is a key regulatory gene that determines fruit spine color in cucumber, and is a good candidate for the *B* (black spine) gene controlling the black fruit-spine trait, which regulates the pigmentation of black spines [197, 198]. A total of 162 *MYB* genes have been identified in watermelon [199].

The GRAS family constitutes one of the major plant-specific TF families that are related to plant growth, development, cell signaling, and stress tolerance [200]. It has been reported that a total of 237 *GRAS* genes were identified in six Cucurbitaceae crop genomes. The number of *GRAS* genes was little different among these species, including *Cucumis sativus* (37), *Cucumis melo* (36), *B. hispida* (35), *Citrullus lanatus* (37), and *Lagenaria siceraria* (37) [201, 202], while the number present in *Cucurbita moschata* (55) was considerably greater. It is known that silencing the *SlGRAS2* gene can reduce fruit weight during tomato fruit development [203]. The study proposed that several genes homologous to *SlGRAS2* (*CmoCh09G009100.1*, *CmoCh01G012140.1*, *MELO3C018144T1*) among these *GRAS* genes might potentially function in fruit development [203].

The NAC domain genes are also one of the largest TF families in plants [204]. A total of 81 genes encoding 92 proteins of the NAC-domain family have been identified in the melon genome [204, 205]. They play an important part in the regulation of fruit ripening in different plants and *CmNAC-NOR*, a melon *NAC* gene family member, is a homolog of tomato *Nor* gene (*SlNAC-NOR*), involved in the climacteric fruit ripening process [136, 204, 206]. The *NAC* gene *SlNAC4* can influence carotenoid accumulation and ethylene synthesis and is a positive regulator of fruit ripening in tomato [206]. The precise roles of the crucial tomato ripening ‘master regulators’, including MADS-RIN, NAC-NOR, and SPL-CNR, have been re-evaluated and it turns out that their severe ripening-inhibition phenotypes result from gain-of-function mutations [136]. Nevertheless, in the wild type, these regulators, plus *Nor-like1* and other *MADS* and *NAC* genes, together with ethylene, play major roles in changes in color, flavor, texture, and ripening progression through promoting the full expression of related genes [206, 207]. *MADS-box* genes have been reported to regulate fruit expansion and ripening processes in melon [205, 208]. In addition, there are many other TFs involved in the regulation of fruit ripening, including the positive regulators TAGL1 [209] and LeHB-1 [210] and the negative regulators LeERF6 [211] and LeAP2a [212].

## Transcriptomics

**Figure 3:**
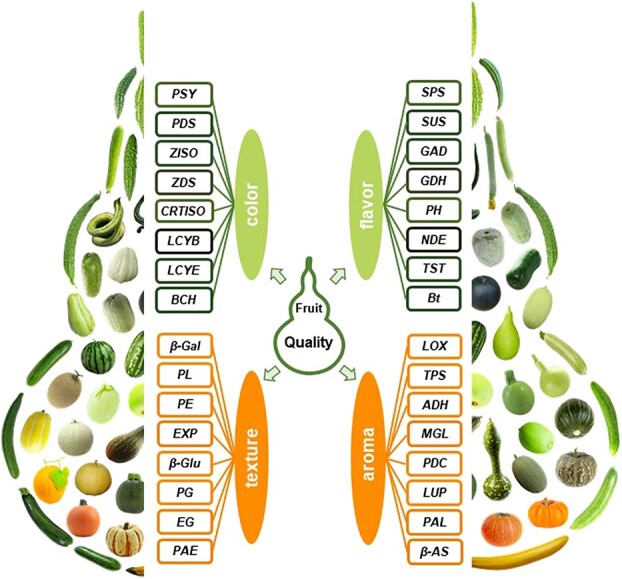
Genes involved in fruit quality in Cucurbitaceae plants

Transcriptome analysis has become an effective approach to understanding the gene networks that govern quality and developmental processes (Fig. 3) and can aid in identifying and exploiting superior cultivars with desirable traits, thus accelerating the Cucurbitaceae plant-breeding process. The transcriptome sequences of many Cucurbitaceae plants, including *Cucurbita maxima* [35], *Cucurbita moschata* [35], *Cucurbita argyrosperma* [35], bottle gourd [30], wax gourd [37], chayote [46], snake gourd [45], watermelon [31, 32], and zucchini [29], are available in the Sequence Read Archive (SRA) database of NCBI. These data provide important information on protein-coding gene prediction, new gene discovery, and gene functional annotation. In addition, transcriptome sequencing has been employed to investigate the molecular basis of the development of many fleshy fruits in Cucurbitaceae species, including cucumber [152, 213–215], melon [216–218], watermelon [219], bitter gourd [150], *Momordica cochinchinensis* [220], bottle gourd [156], zucchini [92, 158, 221], pumpkin [222, 223], wax gourd [224], snake gourd [45], and chayote [46]. Many DEGs related to fruit quality have been identified and Table 3 shows the integrated gene information derived from the transcriptome data of Cucurbitaceae plants.

**Table 3:** Identified DEGs related to fruit quality from transcriptome data in Cucurbitaceae plants.

**Cucurbitaceae**	**Color-related genes**	**Texture-related genes**	**Aroma-, flavor-, and taste-related genes**	**Plant hormone-related genes**	**Key transcription factors**
Cucumber (*Cucumis sativus*)	*β-carotene hydroxylase* (*BCH*), *β-carotene 3-hydroxylase* (*BCH*), *β-carotene isomerase* (*BISO*), *carotenoid cleavage dioxygenase 7* (*CCD7*), *9-cis-epoxycarotenoid dioxygenase* (*NCED*)	*β-galactosidase* (*β-Gal*), *β-glucosidase* (*β-Glu*), *β-amylase 1/3* (*BMY1/3*), *cellulase 3* (*CL3*), *pectin lyase* (*PL*), *pectinesterase* (*PE*), *pectin methylesterase 3* (*PME3*), *pectinacetylesterase* (*PAE*), *xyloglucan:xyloglucosyl transferase* (*XET5*), *expansin* (*EXP*), *cellulose synthase* (*CeSA*)	*terpene synthase 21* (*TPS21*), *lupeol synthase* (*LUS*), *glutamate dehydrogenase 2* (*GDH2*), *lipooxygenase* (*LOX*), *phenylalanine ammonia-lyase 2* (*PAL2*), *alcohol dehydrogenase 1* (*ADH1*), *sucrose synthase 4/5* (*SUS4/5*), *β-amyrin synthase* (*β-AS*)	*1-aminocyclopropane-1-carboxylate synthase 8/10* (*ACS8/10*), *gibberellin-responsive protein*, *histidine phosphotransfer protein* (*HP*), *AUX1*, *Aux/IAA*, *small auxin-up-regulated RNA* (*SAUR*), *ent-kaurenoic acid* (*KAO*), *gibberellin 20-oxidase* (*GA20ox*), *gibberellin 2-oxidase* (*GA2ox*), *gibberellin insensitive dwarf 1* (*GID1*), *auxin-responsive GH3 family protein* (*GH3*), *gibberellin-regulated family protein* (*GASA*), *auxin-responsive protein*, *auxin-induced protein 13*, *ethylene insensitive 3* (*EIN3*), *ethylene response factor* (*ERF*), *gibberellin 2-oxidase 8* (*GA2ox8*)	*AP2/ERF*, *GRAS*, *HSF*, *LFY*, *MADS*, *NAC*, *WRKY*, *YABBY*, *Zinc finger protein*, *bZIP*, *MYB*, *TCP*, *WD40*, *bHLH*, *SBP*, *NF-YA*, *AUX/IAA*
Melon (*Cucumis melo*)	*phytoene synthase* (*PSY*), *carotenoid hydroxylases* (*CYP97A3*), *9-cis-epoxycarotenoid dioxygenases* (*NCED*), *abscisic acid 8′-hydroxylase* (*CYP707A*)	*carotenoid isomerase* (*CRTISO*), *carotenoid hydroxylases* (*CYP97A3*), *9-cis-epoxycarotenoid dioxygenases* (*CCD*), *abscisic acid 8′-hydroxylase* (*CYP707A*)	*sucrose phosphate synthase 2* (*CmSPS2*), *sucrose synthases* (*CmSUS1*, *CmSUS2 and CmSUS-LIKE1*), *hexokinases* (*CmHK2 and CmHK3*), *fructokinase 3* (*CmFK3*), *acid invertases 2* (*CmAIN2*), *cell wall invertases* (*CmCIN2 and CmCIN3*), *phosphoglucose isomerase cyt* (*CmPGIcyt*), *α-galactosidase 2* (*GAL2*), *phosphoenolpyruvate carboxykinases* (*PCK1*, *PCK2 and PCK3*), *cytosolic NADP^+^-dependent isocitrate dehydrogenases* (*cICDH1 and cICDH2*), *lipoamide dehydrogenase 1* (*LPD1*), *malate dehydrogenase* (*MD1*, *MD2*), *aconitate 1* (*ACO1*), *2-oxoglutarate dehydrogenase* (*OGDH*), *pyruvate dehydrogenase* (*PD*)	No data	*AP2/EREBP*, *Constans-like zinc finger*, *C2H2 zinc finger*, *GOLDEN2-like*, *MYB*, *bHLH*, *WRKY*
Watermelon (*Citrullus lanatus*)	*phytoene synthase 1* (*PSY1*), *lycopene β-cyclase* (*LCYB*), *9-cis-epoxycarotenoid dioxygenase 5* (*NCED5*), *β-carotene hydroxylase* (*BCH*), *carotenoid β-ring hydroxylase* (*CYP97A3*), *carotenoid cleavage dioxygenase* (*CCD*)	*pectin methylesterase* (*PME*), *pectinesterase* (*PE*), *proline-rich proteins* (*PRPs*), *fasciclin-like arabinogalactan proteins* (*FLAs*), *xyloglucan endotransglycosylases* (*XET*s), *early nodulin-like proteins* (*ENOD*s), *S-adenosyl methionine decarboxylase* (*SAMDC*), *β-D-glucosidase* (*β-Glu*), *β-galactosidase* (*β-Gal*), *cellulose synthase* (*CeSA*), *endo-1*,*4-β-glucanase* (*EG*), *endoglucanase* (*EG*)	*delta7 sterol C-5 desaturase* (*5-DES*), *phenylalanine ammonia lyase* (*PAL*), *pyruvate decarboxylase* (*PDC*), *malate dehydrogenase* (*MD*), *sucrose synthase* (*SUS*), *sucrose-phosphate synthase* (*SPS*), *ascorbate peroxidase* (*APX*), *squalene synthase* (*SQS*)	*ethylene response factor 1* (*ERF1*), *gibberellin 20-oxidase* (*GA20ox*), *Gibberellin regulated protein* (*GASA*), *auxin-repressed protein ARP1* (*ARP1*), *abscisic acid response protein*, *Aux/IAA protein*, *auxin response factor 2* (*ARF2*), *auxin-repressed protein* (*ARP*)	*AP2/ERF*, *bZIP*, *MADS*, *MYB*, *NAC*, *WRKY*
Bitter gourd (*Momordica charantia*)	*phytoene synthase* (*PSY*), *zeta-carotene desaturase* (*ZDS*), *15-cis-phytoene desaturase* (*PDS*), *lycopene β-cyclase* (*LCYB*), *lycopene epsilon-cyclase* (*LCYE*), *prolycopene isomerase* (*CRTISO*), *carotene epsilon-monooxygenase* (*CYP97C1*), *zeaxanthin epoxidase* (*ZEP*), *violaxanthin de-epoxidase* (*VDE*), *zeta-carotene isomerase* (*ZISO*), *β-carotene 3-hydroxylase* (*BCH*), *xanthoxin dehydrogenase* (*ABA2*), *flavonol synthase* (*FLS*), *chalcone synthase* (*CHS*)	*Pectinesterase* (*PE*), *pectate lyase* (*PL*), *β-galactosidase* (*β-Gal*), *β-amylase* (*BMY*), *β-glucosidase* (*β-Glu*), *α-mannosidase* (*MANA*), *polygalacturonase 2* (*PG2*)	*sucrose synthase* (*SUS*), *phosphoenolpyruvate carboxylase* (*PEPC*), (*3S*)*-linalool synthase* (*TPS14*), *lipoxygenase* (*LOX*), *alcohol dehydrogenase* (*ADH*), *glutamine synthetase* (*GS*), *glutamate decarboxylase* (*GAD*), *pyruvate decarboxylase* (*PDC*), *phenylalanine ammonia-lyase* (*PAL*), *β-amyrin synthase* (*β-AS*)	*1-aminocyclopropane-1-carboxylate synthase* (*ACS*), *gibberellin 2-oxidase* (*GA2ox*), *gibberellin 20-oxidase GA20ox*, *auxin-responsive protein IAA* (*IAA*), *auxin response factor* (*ARF*), *gibberellin receptor GID1* (*GID1*), *abscisic acid receptor PYR/PYL* (*PYR/PYL*), *jasmonic acid-amino synthetase* (*JAR1*), *ethylene receptor* (*ETR*), *serine/threonine-protein kinase CTR1* (*CTR1*), *ethylene-insensitive protein 2/3* (*EIN2/3*), *ethylene-responsive transcription factor 2* (*ERF2*), *small auxin up RNA* (*SAUR*)	*AP2*, *AP2/ERF*, *Dof*, *NAC*, *WRKY*
Bottle gourd (*Lagenaria siceraria*)	No data	*endoglucanase* (*EG*), *expansin* (*EXP*), *galactoside 2-α-L-fucosyltransferase 2* (*FUT2*), *galacturonosyltransferase-like 2* (*GATL2*), *xyloglucan endotransglucosylase/hydrolase 2* (*XTH2*), *xyloglucan glycosyltransferase 5* (*CSLC5*), *xyloglucan endotransglucosylase/hydrolase protein* (*XTH*)	*sucrose-phosphate synthase 4* (*SPS4*), *sucrose synthase 5* (*SUS5*)	*auxin-binding protein ABP20*, *auxin-induced protein*, *auxin efflux carrier component 8*, *cytokinin dehydrogenase* (*CKX*), *gibberellin 3-β-dioxygenase 1* (*GA3ox1*)	*APRR2*, *bHLH*, *bZIP*, *ERF*, *MYB*, *MYC*, *RAD*, *TFIIB*, *WRKY*
Zucchini (*Cucurbitapepo*)	*phytoene synthase* (*PSY*), *phytoene desaturase* (*PDS*), *β-carotene hydroxylase* (*BCH*), *carotenoid cleavage dioxygenase* (*CCD*), *carotenoid isomerase* (*CRTISO*), *zeta-carotene desaturase* (*ZDS*), *chalcone synthase 2* (*CHS2*), *zeaxanthin epoxidase* (*ZEP*)	*β-amylase 1* (*BMY1*), *pectate lyase-like* (*PL*), *β-glucosidase* (*β-Glu*), *β-galactosidase* (*β-Gal*), *β-1*,*3-glucanase 3* (*GLU3*), *expansin* (*EXP*), *endoxyloglucan transferase a2* (*xrt2*), *pectinesterase* (*PE*), *pectate lyase 12* (*PL12*), *polygalacturonase* (*PG*), *glucan endo-1*,*3-β-D-glucosidase* (*BGL2*), *cellulose synthase-like protein D5* (*CSLD5*)	*linoleate 9S-lipoxygenase 6* (*LOX6*), *glutamate dehydrogenase 2* (*GDH2*), *linoleate 13S-lipoxygenase 2–1* (*LOX*), *alcohol dehydrogenase* (*ADH*), *granule-bound starch synthase* (*GBSS*), *sucrose synthase 5* (*SUS5*)	*1-aminocyclopropane-1-carboxylate oxidase 3/4* (*ACO3/4*), *ethylene insensitive 2/3* (*EIN2/3*), *ethylene response 1/2* (*ETR1/2*), *ethylene response factor* (*ERF*), *1-aminocyclopropane-1-carboxylate synthase* (*ACS*), *gibberellin 2-oxidase* (*GA2ox*), *auxin-induced protein*, *auxin response factor* (*ARF*), *auxin-responsive protein IAA* (*IAA*), *gibberellin-regulated protein 9* (*GASA9*), *auxin-induced protein AUX28*, *abscisic acid 8′-hydroxylase* (*CYP707A*), *auxin-responsive protein SAUR72*, *abscisic acid receptor PYL2*, *cytokinin hydroxylase-like*	*MYB*, *bHLH*, *AUX/IAA*, *AP2*, *AP2/ERF*, *SBP*, *CAAT*, *HSF*, *MBF1*, *bZIP*, *NAC*, *MADS*, *GRF*, *WRKY*
Pumpkin (*Cucurbita maxima*)	*phytoene synthase* (*PSY*), *15-cis-phytoene desaturase* (*PDS*), *zeta-carotene isomerase* (*ZISO*), *prolycopene isomerase* (*CRTISO*), *lycopene epsilon-cyclase* (*LCYE*), *lycopene β-cyclase* (*LCYB*), *β-ring hydroxylase* (*CYP97A3*), *β-carotene 3-hydroxylase* (*BCH*), *carotene epsilon-monooxygenase* (*CYP97C1*), *zeaxanthin epoxidase* (*ZEP*), *violaxanthin de-epoxidase* (*VDE*), *chalcone synthase* (*CHS*)	*endoglucanase* (*EG*), *expansin* (*EXP*), *xyloglucan endotransglucosylase/hydrolase protein* (*XTH*), *β-amylase 1* (*BMY1*), *pectate lyase-like* (*PL*), *pectin acetylesterase* (*PAE*), *pectinesterase* (*PE*), *β-glucosidase* (*β-Glu*), *β-galactosidase* (*β-Gal*), *polygalacturonase* (*PG*), *glucan endo-1*,*3-β-glucosidase* (*BG*), *cellulose synthase* (*CeSA*)	*alcohol dehydrogenase* (*ADH*), *β-fructofuranosidase* (*INV*), *fructokinase* (*FK*), *hexokinase* (*HK*), *glucose-6-phosphate isomerase* (*PGI*), *phosphoglucomutase* (*PGM*), *UTP-glucose-1-phosphate uridylyltransferase* (*UGPase*), *sucrose synthase* (*SUS*), *sucrose-phosphate synthase* (*SPS*), *ADP-sugar diphosphatase* (*NUDX14*), *glucose-1-phosphate adenylyltransferase* (*AGPase*), *1*,*4-α-glucan branching enzyme* (*SBE*), *trehalose 6-phosphate synthase/phosphatase* (*TPS*), *α-amylase* (*amyA*), *4-α-glucanotransferase* (*malQ*), *UDP-glucose 6-dehydrogenase* (*UGDH*), *UDP-glucuronate decarboxylase* (*UXS1*), *1*,*4-β-D-xylan synthase* (*XS*), *β-D-xylosidase 4* (*XYL4*), *UDP-glucuronate 4-epimerase* (*GAE*), *α-1*,*4-galacturonosyltransferase* (*GAUT*)	*1-aminocyclopropane-1-carboxylate oxidase* (*ACO*), *1-aminocyclopropane-1-carboxylate synthase* (*ACS*), *ethylene insensitive 2/3* (*EIN2/3*), *ethylene-responsive transcription factor* (*ERF*), *ethylene receptor 1* (*ETR1*), *ethylene response sensor 1* (*ERS1*), *gibberellin 2-oxidase* (*GA2ox*), *gibberellin 20-oxidase* (*GA20ox*), *auxin-induced protein*, *auxin response factor 6* (*ARF6*), *auxin-responsive protein IAA* (*IAA*), *gibberellin-regulated protein* (*GASA*), *abscisic acid 8′-hydroxylase* (*CYP707A*)	*bHLH*, *AP2/ERF*, *MYB*, *NAC*, *AUX/IAA*, *bZIP*, *WRKY*
Snake gourd (*T. anguina*)	*phytoene synthase* (*PSY*), *15-cis-phytoene desaturase* (*PDS*), *prolycopene isomerase* (*CRTISO*), *zeta-carotene desaturase* (*ZDS*), *lycopene β-cyclase* (*LCYB*), *lycopene epsilon cyclase* (*LCYE*), *15-cis-zeta-carotene isomerase* (*ZISO*), *9-cis-epoxycarotenoid dioxygenase NCED2* (*NCED2*), *β-carotene 3-hydroxylase 1* (*BCH1*), *carotene epsilon-monooxygenase* (*CYP97C1*)	*cellulose synthase-like protein E1* (*CSLE1*), *endoglucanase* (*EG*), *expansin* (*EXP*), *glucan 1*,*3-β-glucosidase* (*BGL*), *pectinesterase* (*PE*), *polygalacturonase* (*PG*), *β-galactosidase* (*β-Gal*), *glucan endo-1*,*3-β-D-glucosidase* (*BGL2*), *pectin acetylesterase* (*PAE*), *pectin methyltransferase* (*PMT*), *β-glucosidase* (*β-Glu*), *pectate lyase* (*PL*), *glucan endo-1*,*3-β-glucosidase* (*BG*)	*linoleate 9S-lipoxygenase-like* (*LOX*), *linoleate 13S-lipoxygenase 2–1*(*LOX*), *alcohol dehydrogenase* (*ADH*), *glutamate dehydrogenase 1* (*GDH1*), *phenylalanine ammonia-lyase* (*PAL*), *sucrose synthase* (*SUS*)	*1-aminocyclopropane-1-carboxylate oxidase* (*ACO*), *ethylene-responsive transcription factor* (*ERF*), *ethylene receptor 2* (*ETR2*), *abscisic acid receptor PYR1-like*, *auxin-induced protein*, *auxin-responsive protein IAA*, *auxin response factor* (*ARF*), *abscisic acid receptor PYR1*, *auxin-responsive protein SAUR50-like*, *gibberellin-regulated protein* (*GASA*), *gibberellin receptor*, *abscisic acid 8-hydroxylase 4-like*, *gibberellin 2-β-dioxygenase 1-like*, *gibberellin 3-oxidase 3* (*GA3ox3*), *auxin transporter-like protein* (*LAX*), *auxin-responsive protein SAUR71-like*, *ethylene insensitive 3* (*EIN3*), *ethylene response sensor 1* (*ERS1*)	*AP2/ERF*, *MYC*, *ERF*, *ANT*
Chayote (*Sechium edule*)	*phytoene synthase* (*PSY*), *9-cis-epoxycarotenoid dioxygenase NCED2/3* (*NCED2/3*), *zeaxanthin epoxidase* (*ZEP*), *carotenoid 9*,*10-cleavage dioxygenase 1* (*CCD1*), *carotenoid cleavage dioxygenase 4/8* (*CCD4/8*), *β-carotene hydroxylase 2* (*BCH2*), *β-carotene isomerase D27* (*D27*), *flavonol synthase* (*FLS*), *chalcone synthase 2* (*CHS2*)	*expansin* (*EXP*), *glucan endo-1*,*3-β-glucosidase* (*BG*), *polygalacturonase* (*PG*), *xyloglucan endotransglucosylase/hydrolase* (*XTH*), *β-galactosidase* (*β-Gal*), *β-glucosidase* (*β-Glu*), *cellulose synthase* (*CeSA*), *endoglucanase* (*EG*), *pectin acetylesterase* (*PAE*), *pectinesterase* (*PE*), *α-mannosidase* (*MANA*), *β-amylase 1* (*BMY1*), *pectin methyltransferase* (*PMT*), *pectinesterase/pectinesterase inhibitor 25* (*PME25*), *pectate lyase* (*PL*)	*linoleate 13S-lipoxygenase 2–1* (*LOX*), *linoleate 13S-lipoxygenase 3–1* (*LOX*), *linoleate 9S-lipoxygenase* (*LOX*), *alcohol dehydrogenase-like 6* (*ADH6*), *glutamate dehydrogenase 2* (*GDH2*), *terpene synthase 10* (*TPS10*), *sucrose-phosphate synthase 1* (*SPS1*), *sucrose synthase* (*SUS*), *sucrose-phosphatase 1* (*SPP1*)	*1-aminocyclopropane-1-carboxylate synthase* (*ACS*),*1-aminocyclopropane-1-carboxylate oxidase* (*ACO*), *ethylene-responsive transcription factor* (*ERF*), *abscisic acid 8-hydroxylase* (*CYP707A*), *auxin response factor* (*ARF*), *auxin-induced protein*, *auxin-responsive protein IAA*, *gibberellin receptor GID1B*, *abscisic acid receptor PYL8/9*, *auxin-responsive protein SAUR*, *gibberellin 20 oxidase 1* (*GA20ox1*), *gibberellin 2-β-dioxygenase* (*GA2ox*), *gibberellin-regulated protein 14* (*GASA14*)	*AP2*, *MYB*, *NAC*, *WRKY*, *MYC*, *bHLH*, *SBP*, *AP2/ERF*, *bZIP*, *GRF*

## Importance of genome resequencing for the development of molecular breeding

Whole-genome resequencing technology has been used to investigate wide germplasm resources. Resequencing of multiple materials from different crop species has helped reveal the domestication history of cucurbit crops and candidate genes or loci influencing agronomic traits. Important cucurbit crops that have been resequenced include *Citrullus lanatus* [31, 32], *Cucumis sativus* [15], *Cucumis melo* [24, 25, 225], *B. hispida* [37], *M. charantia* [41], and *Lagenaria siceraria* [8]. Resequencing and provision of large-scale germplasm resources can be applied to population genomic analyses and GWAS to identify QTLs. Genome-wide single-nucleotide polymorphism (SNP) markers have been widely used in molecular breeding for mapping of important fruit quality trait genes and can contribute to the discovery of candidate loci or key genes and molecular markers associated with important traits in cucurbits for crop improvement (Table 4).

**Table 4:** List of resequenced species of Cucurbitaceae plants.

**Date**	**Species**	**Number of accessions**	**Average sequencing depth**	**SNPs**	**Indels**	**Reference**
2012	*Citrullus lanatus*	20	5–16×	6 784 860	965 006	31
2013	*Cucumis sativus*	115	18.3×	3 305 010	336 081	15
2019	*Cucumis melo*	1175	4.71–18.92×	5 678 165	957 421	25
2019	*Citrullus lanatus*	414	14.5×	19 725 853	6 675 290	32
2019	*B. hispida*	146	15.68×	16 183 153	2 190 214	37
2019	*Cucumis melo*	50	18×	1 761 822	735 486	24
2020	*M. charantia*	60	-	6 135 286		41
2020	*M. charantia*	189	8.8–37.8	14 450 193	1 588 578	42
2021	*Lagenaria siceraria*	50		2 004 276	399 664	8
2021	*Cucumis metuliferus*	40	12×	1 688 333	247 969	28

A genome variation map for cucumber fruit was obtained through deep resequencing of 115 cucumber lines and a region containing a gene related to the loss of bitterness in cucumber fruit was identified [15]. The QTL mapping of cucumber also identified eight QTLs related to leaf size or fruit length [15]. Moreover, a natural genetic variant in a *β-carotene hydroxylase 33* gene (*CsaBCH1*) that resulted in accumulation of β-carotene and formation of orange fruit endocarp was identified, which could be helpful in obtaining varieties with higher nutritional value [15]. In Payzawat melon, six structural gene variants potentially controlling the thickness of the epidermis were identified by analyzing the QTLs related to epidermis thickness [24]. In addition, Zhao *et al*. [25] reported a comprehensive map of the melon genomic variation that originated from the resequencing of 1175 accessions, and GWAS studies for 16 agronomic traits identified 208 loci markedly related to fruit quality, mass, and morphological characters. This study proposed that the strong differentiation between *Cucumis melo* and *Cucumis agrestis* may contribute to breeding. Watermelon breeding has mainly focused on fruit quality traits, particularly, sweetness, flesh color, and rind pattern, which has led to the narrow genetic base of watermelon [32]. In 2013, Guo *et al*. [31] resequenced 20 watermelon accessions and identified many disease-resistance genes that had been lost during domestication. Thus, improving resistance to pathogens is an ongoing goal of sweet watermelon breeding programs. Interestingly, *Citrullus amarus, Citrullus colocynthis*, and *Citrullus mucosospermus* have been used for breeding studies to find new sources of disease and insect resistance to improve sweet watermelon. Whole-genome resequencing of 414 accessions identified genomic regions associated with critical fruit quality traits and using GWAS identified a total of 43 association signals, which provided useful information for watermelon breeding [32].

Bitter gourd is an important vegetable and medicinal plant in the Cucurbitaceae family. The bitter taste of bitter gourd is due to the existence of cucurbit triterpenoid compounds cucurbitacins [42] and it has the potential for further improvement [41]. A total of 1507 marker loci were genotyped by using restriction-associated DNA tag sequencing (RAD-seq) analysis, resulting in an improved linkage map [6]. A total of 255 scaffolds were assigned to the linkage map through anchoring RAD tag markers [6]. Interspecific crosses play a vital part in *Cucurbita* breeding for transferring favorable traits between species [34], and 40 transcriptomes assembled for 11 species of the *Cucurbita* genus could serve as a valuable source of molecular markers [29]. In addition, research on the resequencing of 146 wax gourd accessions mapped nine QTLs for fruit-size-associated traits, and 11 candidate domestication genes and a number of genomic regions putatively related to the determination of wax gourd fruit size have been identified using GWAS [37]. These resequencing results and the genome sequences presented will provide a basis for DNA marker development, gene identification, and molecular breeding of these Cucurbitaceae species.

## Future prospects

New-generation sequencing technologies and breeding techniques, together with bioinformatics tools, have greatly promoted the progress of plant breeding, although further bioinformatics analysis of whole-genome sequencing data of Cucurbitaceae crops is still required in order to accelerate Cucurbitaceae crop breeding improvement. The integration of multiomics data with genetic and phenotypic data will help to identify genes related to important traits and accelerate the process of plant breeding [71].

Genetic transformation and genome editing of Cucurbitaceae plants have a significant development potential for obtaining new cucurbit phenotypes with ideal traits. A reverse genetic approach, Targeting Induced Local Lesions in Genomes (TILLING), can be applied to the breeding of Cucurbitaceae crops and help to improve agronomic traits [226]. Different DNA mutant TILLING libraries have been set up in cucurbits [227–231]. This approach has provided a resource for plant breeding programs and future functional genomics study. Genome editing technology is attracting attention and breeding efficiency can be rapidly improved through combining the genomic and variomic information on crops [232]. Developing efficient and reliable genetic transformation technology for the target crops will contribute to the wide application of this approach in Cucurbitaceae crops. CRISPR/Cas9 is a common and efficient technique for genome editing and has been used for Cucurbitaceae crops to knock out target genes and obtain crop materials with desirable agronomic traits [233, 234], such as cucumber [235, 236], watermelon [237, 238], and pumpkin [239], and has become a precision-breeding approach for modifying traits in plants species [240]. In the future, a wide range of genome analysis and editing research is expected to expand our understanding and implementation for Cucurbitaceae plant breeding programs.
